# Supplementary material for: Reconciling Mining with the Conservation of Cave Biodiversity: A Quantitative Baseline to Help Establish Conservation Priorities
Source: PLoS One. 2016 Dec 20;11(12):e0168348. doi: 10.1371/journal.pone.0168348 (PMC5173368; doi:10.1371/journal.pone.0168348)
Supplement: S1 Dataset — (ZIP) [file pone.0168348.s002.zip › Taxa/Serra Norte/SN_2013/N4E/Lista N4E-86.pdf]

CAVIDADE N4E-0086

Classe	Ordem	Fam/Outros	Gên/Outros	Espécie	S-U	U-S	Zon.
Arachnida	Acari	Cunaxidae		sp.2		X	E
Arachnida	Acari	Eupodidae		sp.1		X	E
Arachnida	Acari	Laelapidae		sp.3	X		E
Arachnida	Acari	Trombiculidae		sp.41	X		E
Arachnida	Araneae	Araneidae	*Alpaida*	aff. *negro*	X	X	E
Arachnida	Araneae	Caponiidae		jovem	X		E
Arachnida	Araneae	Corinnidae		jovem	2		E
Arachnida	Araneae	Drymusidae	*Drymusa*	*spelunca*	x	X	E
Arachnida	Araneae	Ochyroceratidae		jovem		X	E
Arachnida	Araneae	Pholcidae	*Mesabolivar*	sp.1	x	X	E
Arachnida	Araneae	Salticidae		jovem	X		E
Arachnida	Araneae	Symphytognathidae	*Anapistula*	sp.1	X	X	E
Arachnida	Araneae	Theridiidae	*Thymoites*	sp.1	X	X	E
Arachnida	Araneae	Theridiosomatidae	*Plato*	sp.1	X	X	E
Arachnida	Opiliones	Cosmetidae	*Roquettea*	*singularis*	1		E
Arachnida	Opiliones	Escadabiidae		sp.2	X	X	E
Arachnida	Opiliones	Stygnidae	*Protimesius*	*laevis*	1		E
Arachnida	Opiliones	Stygnidae	*Protimesius*	*gracilis*	3		E
Chilopoda	Scolopendromorpha	Scolopocryptopidae		sp.3	1		E
Chilopoda	Scutigeromorpha	Pselliodidae		jovem	1	3	E
Diplopoda	Polydesmida	Chelodesmidae		sp.3		1	E
Diplopoda	Polydesmida	Pyrgodesmidae		sp.1	X	X	E
Entognatha	Collembola	Entomobryidae		sp.7	X		E
Entognatha	Collembola	Paronellidae		sp.1	X	X	E
Entognatha	Collembola	Paronellidae		sp.4		X	E
Entognatha	Diplura	Campodeidae		sp.1		X	E
Insecta	Blattodea			jovem	1	2	E
Insecta	Coleoptera	Scydmaenidae		sp.6	X		E
Insecta	Diptera	Chironomidae		sp.	X		E
Insecta	Diptera	Dolichopodidae		sp.	X		E
Insecta	Diptera	Lauxaniidae		sp.		X	E
Insecta	Diptera	Psychodidae	Phlebotominae	sp.	X	X	E
Insecta	Diptera	Sciaridae		sp.	X		E
Insecta	Diptera	Tipulidae	Tipulinae	sp.	X	X	E
Insecta	Heteroptera	Reduviidae	Emesinae	sp.	1		E
Insecta	Heteroptera	Reduviidae	Triatominae	sp.		1	E
Insecta	Hymenoptera	Formicidae	*Camponotus*	sp.2		X	E
Insecta	Hymenoptera	Formicidae	*Gnamptogenys*	sp.1	X	X	E
Insecta	Hymenoptera	Formicidae	*Hypoponera*	sp.1		X	E
Insecta	Hymenoptera	Formicidae	*Odontomachus*	sp.1		X	E
Insecta	Hymenoptera	Formicidae	*Pachycondyla*	sp.1	X		E
Insecta	Hymenoptera	Formicidae	*Pachycondyla*	sp.2		X	E
Insecta	Hymenoptera	Formicidae	*Pheidole*	sp.3	X		E
Insecta	Lepidoptera	Noctuoidea		sp.2		1	E
Insecta	Lepidoptera	Noctuoidea		sp.5		2	E
Insecta	Orthoptera	Mogoplistidae	Mogoplistinae	jovem		3	E
Insecta	Orthoptera	Phalangopsidae	*Paraclodes*	jovem	4		E
Insecta	Orthoptera	Phalangopsidae	*Phalangopsis*	jovem	3	7	E
Insecta	Orthoptera	Podoscirtidae		sp.		1	E

Insecta	Psocoptera	Epipsocidae	*Mesepipsocus*	sp.1	X	X	E
Symphyla		Scutigerellidae		sp.		X	E
Amphibia	Anura	Strabomantidae	*Pristimantis*	cf. *fenestratus*	2	3	E
Mammalia	Chiroptera	Emballonuridae	*Peropteryx*	*kappleri*	2	1	E
Mammalia	Rodentia	Cricetidae	*Rhipidomys*	sp.		2	E
